# Energy expenditure related biomarkers following bariatric surgery: a prospective six-month cohort study

**DOI:** 10.1186/s12893-024-02421-3

**Published:** 2024-04-27

**Authors:** Mahsa Hatami, Mohammad Hassan Javanbakht, Neda Haghighat, Zahra Sohrabi, Rahman Yavar, Abdolreza Pazouki, Gholamreza Mohammadi Farsani

**Affiliations:** 1 Department of Clinical Nutrition, School of Nutritional Sciences and Dietetics, Tehran University of Medical Sciences (TUMS), Tehran, Iran; 2 Minimally Invasive Surgery Research Center, Iran University of Medical Sciences, Tehran, Iran; 3 Department of Cellular and Molecular Nutrition, School of Nutritional Sciences and Dietetics, Tehran University of Medical Sciences (TUMS), Tehran, Iran; 4 Laparoscopy Research Center, Shiraz University of Medical Sciences, Shiraz, Iran; 5 Department of Community Nutrition, School of Nutrition and Food Sciences, Shiraz University of Medical Sciences, Shiraz, Iran; 6 Department of Genetics, Akbar-Abadi Hospital, Iran University of Medical Sciences, Tehran, Iran; 7 Center of Excellence of International Federation for Surgery of Obesity, Hazrat-E Rasool Hospital, Tehran, Iran

**Keywords:** Bariatric Surgery, PGC-1α, UCP-2, Body Composition, Mitochondrial Biogenesis, Energy Expenditure, Thermogenesis

## Abstract

**Background:**

Mitochondria dysfunction is one of the major causes of insulin resistance, and other countless complications of obesity. PGC-1α, and UCP-2 play key roles in energy expenditure regulation in the mitochondrial thermogenesis. However, the effects of bariatric surgery on the level of PGC-1α and UCP-2 and their relationships are unclear.

**Objective:**

This study aimed to investigate the effect of bariatric surgery on key pathways in energy, and to assess the potential predictive role of body composition and metabolic parameters in this regard.

**Settings:**

Hazrat-e Rasool General Hospital, Center of Excellence of International Federation for Surgery of Obesity.

**Methods:**

This prospective cohort study was carried out on 45 patients with morbid obesity who underwent Roux-en-Y gastric bypass surgery. The patients have evaluated three-time points at baseline, three, and six months after the surgery. Body composition components, the levels of PGC-1α, UCP-2, and metabolic parameters were measured three times during this study.

**Results:**

Significant changes in TWL%, EBMIL%, and metabolic lab tests were observed at three- and six months post-surgery (*P* < 0.001). The PGC-1α and UCP-2 had a significant increase three and then six-month post-operation compared with the baseline (*P* < 0.001). Moreover, multivariate linear regression analysis identified that the changing trend of PGC-1α was associated with insulin, uric Acid, HOMA-IR, fat mass and trunk fat mass. UCP-2 was associated with TSH, AST, fat mass and FFM.

**Conclusions:**

Bariatric surgery has been shown to have a positive effect on UCP-2 and PGC-1α levels, as well as body composition and metabolic parameters. As a result, it is believed that bariatric surgery could improve thermogenesis and energy expenditure by enhancing mitochondrial biogenesis and function. However, further studies are needed to fully understand the precise mechanisms and possible causal relationship.

## Introduction

Bariatric surgery is considered an effective way for weight loss in the short and long term. It can help patients decrease the risk of obesity-related diseases [1, 2]. There are strong evidences supporting the safety and effectiveness of bariatric surgeries [3]**.** Most pathologic co-morbidities associated with obesity including diabetes, hypertension, sleep apnea, and dyslipidemia could improve after the bariatric surgery. Further, these surgeries could improve life expectancy and also the quality of life [4]. Mechanisms behind the effects of bariatric surgery on weight loss are poorly understood. Most of the studies focused on the changes in the brain-gut axis and neuropeptides or factors such as ghrelin, NPY, glucagon-like growth factor, and so on. All of the aforementioned changes could finally regulate food intake, appetite, glucose metabolism, and reduce liver gluconeogenesis [5]. There aren’t enough studies assessing the cellular or molecular mechanisms behind the effects of bariatric surgery on weight and energy expenditure status. Fat accumulation in obese people could cause inflammation and mitochondrial dysfunction which could in turn decrease mitochondrial biogenesis [6]. Downregulation of mitochondrial biogenesis and dysfunction in mitochondrial oxidation caused by adipocyte dysfunction and also macrophage infiltration could induce insulin resistance, metabolic syndrome, and liver steatosis [7]. One of the proteins involved in the regulation of mitochondrial biogenesis is Peroxisome proliferator-activated receptor coactivator-1α (PGC-1α) [8]. This protein could induce energy production in the electron transport chain in the mitochondria [9]. Changes in the PGC-1α were not assessed in the previous studies of post-bariatric surgery. Moreover, another mitochondrial protein called uncoupling protein-2 (UCP-2) plays a pivotal role in the optimal mitochondrial function and thermogenesis, and this way it can affect body weight and insulin resistance as well [10]. Any increase in the level of PGC-1α and UCP-2 in the white adipose tissue could demonstrate the characteristics of increasing fat oxidation and thermogenesis by upregulation of mitochondrial biogenesis and function [11]. However, few studies assess the changes in PGC-1α and UCP-2 post-operatively, and due to the important role of mitochondrial dysfunction in obesity pathogenesis or lack of success in non-surgical weight loss programs, the aim of this study was to evaluate the changes in serum PGC-1α and UCP-2 simultaneously with the changes in weight and body composition, insulin resistance, blood lipids, insulin, and glycemic control, liver enzymes, and their relationships.

## Material and methods

### Ethics statement

This study was approved by the Ethics Committee of Tehran University of Medical Sciences, Tehran, Iran (Ethics number: IR.TUMS.MEDICINE.REC.1400.011). The details of the study were explained to participants with an explanatory letter and written informed consent was obtained from all participants prior to entering the project.

### Study design, setting and participants

This study was a prospective cohort study that was carried out from December 2022 to July 2023 on 45 patients with morbid obesity with BMI between 40 and 50 kg/m2, aged from 20 to 50 years, who underwent bariatric surgery. All patients were operated on Hazrat-e Rasool General Hospital, Tehran (Center of Excellence of International Federation for Surgery of Obesity) by a particular surgeon (A.P). Roux-en-Y gastric bypass (a 25–30 mL pouch and 130–150 cm Roux-en-Y limb) were performed using the laparoscopic technique with a five-port approach. The Inclusion criteria were willingness to participate in the study and not participating in a specific diet program (for example ketogenic diet, vegetarian, intermittent fasting, etc.) or professional physical activity other than the usual postoperative protocol before and during six months after the surgery. Moreover, this study included those patients without reversal bariatric surgery or previous bariatric surgery, pregnancy, lactation, smoking (At least five cigarettes per day for the past six months), kidney disease, pancreatitis, cancer, uncontrolled thyroid disorders, inflammatory, neurological, and autoimmune diseases and a history of heart attacks and strokes. This study excluded patients who withdrew from surgery or cooperation in the study or were pregnant during the follow-up. All patients were assessed for clinical, anthropometrical, and biochemical variables for three and six months post-operatively.

### Variables, data sources/measurement

#### Anthropometric measurements

Height was measured using a measuring tape attached to a wall and without shoes with a nearest 0.5 cm. A bio-impedance analysis scale (BIA) (TANITA BC-418 BIA) was used to measure body weight, body fat percentage (%), body fat (kg), FFM (Fat-free mass) (kg), muscle mass(kg), trunk Fat (%), trunk fat (kg), trunk FFM (kg), trunk muscle mass (kg), body water (%). Furthermore, Visceral fat level (ranges: 1–59) was measured through BIA, which its validity for the prediction of metabolic syndrome was assessed and confirmed in recent study [12] For accurate results, it was recommended that the patients stay hydrated, that is, drink 1–2 glasses of water three hours before the test and avoid tea, coffee, and alcohol consumption and physical activity 8 h before the BIA. Weight loss was assessed by the %total weight loss (TWL), % excess weight loss (EWL), %excess BMI loss (%EBMIL), and body mass index changes (ΔBMI). %TWL was calculated as [(preoperative weight- weight on follow up)/preoperative weight] × 100. %EWL was calculated as [(preoperative weight- weight on follow-up)/ (preoperative weight − ideal body weight)] × 100. %EBMIL was calculated using the formula: [ 100 * (Initial BMI- Postoperative BMI) / (Initial BMI—25)], moreover, ΔBMI was calculated as (Initial BMI- Postoperative BMI). Ideal weight was defined as the weight corresponding to a BMI of 25 kg/m^2^ [13].

#### Laboratory measurement

Fasting blood samples (10 ml) were collected from all participants for biochemical assessments.: Fasting blood samples (10 ml) were taken at baseline and end of each study phase in the early morning after an overnight fast. Blood samples were immediately centrifuged (Hettich D-78532, Tuttlingen, Germany) at 3500 rpm for 10 min to separate serum. Then, the samples were stored at -80 ^0^C before analysis at the laboratory. ELISA kits (Human ELISA Kit, Shanghai Crystal Day Biotech Co., Ltd) were used to measure the levels of UCP-2, and PGC-1α. All measurements were performed using the ELISA method. Besides, preoperative serum laboratory parameters, including serum fasting blood sugar (FBS), insulin, HbA1c (%), triglyceride, cholesterol, high-density lipoprotein (HDL), low-density lipoprotein (LDL), alanine transaminase (ALT), aspartate aminotransferase (AST), uric acid, and thyroid-stimulating hormone (TSH) were recorded.

#### Post-operative care

All patients were asked to refer to the obesity clinic for multiple assessments of body composition, weight loss, and collection of blood samples, at one, three, and six months after the surgery. According to the international bariatric surgery guidelines, daily multivitamins and mineral supplementation and twice-a-day calcium citrate (500 mg) supplementation were started from the first day after the surgery. Likewise, intramuscular neurobion (including 1000 μg of vitamin B12, 100 mg of vitamin B1, and 100 mg of vitamin B6), and oral vitamin D3 (50,000 IU), were administered post-surgery every month, for all patients. The diet protocol for all patients who undergo bariatric surgery were explained during the regular visits after the surgery. The first two days: clear liquid diet, the second to the fourteenth day: Full liquid diet, the 14th to 28th day: soft diet, the 28th day to 1.5 months after the surgery: Puree diet, and 1.5 months post-operation: the usual table food. All patients are advised to participate in physical activity according to the guidelines set by the American Heart Association / American College of Sports Medicine. These guidelines suggest engaging in 30 min of moderate-intensity aerobic activity for five days per week, or 20 min of vigorous-intensity activity for three days per week, to enhance weight loss and preserve muscle mass.

### Statistical methods

The normal distribution of the variables was checked using Kolmogorov–Smirnov test. Continuous variables were shown as means and standard error. Repeated measures ANOVA analysis was performed to assess the time-by-surgery interaction effect on each outcome Multivariate linear regression models were used to predict the association between the mean changes of PGC-1α/ UCP-2 and other clinical variables including anthropometric indices, body composition measurements, and biochemical parameters. Age, Sex, and BMI was adjusted in multivariable Analysis as covariates. The *P*-value of less than 0.05 was considered statistically significant and analysis was performed by SPSS 26.0.

## Results

### Characteristics of the participants

Out of 53 referred patients with morbid obesity, 45 patients, whose mean age was 40.47 ± 9.6 years, mean BMI was 44.66 ± 3.62, and 91.1% were female enrolled in the present study (Table 1). During the 6 months follow-up of the study, 8 patients left the study due to different reasons including starting an unplanned exercise regimen, being unable to maintain their planned diets, immigration, and being unable to do laboratory examination. Of the study participants, 15 patients (33.3%) had T2DM, 13 (28.8%) were hypertensive, 19 (42%) had Dyslipidemia, 10 (22.2%) had Hypothyroidism, and 17 (37.8) had polycystic ovary syndrome (PCOS). Preoperative characteristics of the patients are demonstrated in Table 1, which shows that age, sex, anthropometric indices, biochemical parameters, associated medical problems, and food habits.

**Table 1:** Baseline before surgery characteristics of patients

Variables	Value
Age, mean ± SD, Year	40.47 ± 9.6
Female Sex, no (%)	41 (91,1)
Weight, mean ± SD, Kg	119.65 ± 20.71
Height, mean ± SD, Cm	163.1 ± 9.9
BMI, mean ± SD, Kg/m2	44.66 ± 3.62
FBS, mean ± SD, mg/dL	108.18 ± 25.96
HbA1c, mean ± SD, %	5.76 ± 0.71
Insulin, mean ± SD, µIU/mL	18.60 ± 7.34
HOMA- IR, mean ± SD	5.12 ± 2.97
ALT, mean ± SD, IU/L	29.05 ± 13.05
AST, mean ± SD, IU/L	30.93 ± 16.50
Uric Acid, mean ± SD	5.17 ± 1.07
TSH, mean ± SD, mIU/L	2.11 ± 1.21
Cholesterol, mean ± SD, mg/dL	183.8 ± 37.84
HDL, mean ± SD, mg/dL	46.81 ± 11.79
LDL, mean ± SD, mg/dL	108.07 ± 30.28
TG, mean ± SD, mg/dL	160.98 ± 52.88
**Comorbidities, no (%)**	
T2DM	15 (33,3)
HTN	13 (28.8)
Dyslipidemia	19 (42)
Hypothyroidism	10 (22,2)
PCO	17 (37.8)
**Food Habits, no (%)**	
Volume Eating	36 (80)
Emotional Eating	26 (57.8)
Grazing	30 (66.7)
Sweet Eating	28 (62.2)

*BMI* Body Mass Index, *FBS* Fasting Blood Sugar, *HbA1c* Hemoglobin A1c, *HDL* High-density lipoprotein, *LDL* Low-density lipoprotein, *PTH* Parathyroid hormone, *AST* Aspartate Transaminase, *ALT* alanine aminotransferase, *TG* triglyceride

### Weight loss at four time points before and after the surgery

The present study measured 4 time points of weight loss, before and after the surgery. In comparison to the preoperative level, weight, and BMI both decreased significantly at 1, 3, and 6 months following the bariatric surgery (*P* < 0.001). The TWL% was 10.22 ± 0.27 in 1st month, 19.15 ± 0.39 in 3rd month and 27.49 ± 0.74 in 6 months after the surgery, which were significantly different over the three periods of testing (*P* < 0.001). The EWL% was 23.75 ± 0.78 in 1st month, 44.44 ± 1.23 in 3rd month, and 63.7 ± 2.06 in 6 months after the surgery which was significantly different over the three periods of testing (*P* < 0.001). The other weight loss indicators including EBMIL% and ΔBMI demonstrated in Table 2.

**Table 2:** Weight loss outcomes at four time points before and after surgery

Variables^a^	**Before surgery**	**After Surgery**			***P*-Value**
	**Baseline**	**1 months**	**3 months**	**6 months**	
Weight	119.65 ± 3.09	107.47 ± 2.85	96.73 ± 2.54	86.67 ± 2.29	< 0.001
BMI	44.67 ± 0.54	40.11 ± 0.52	36.12 ± 0.49	32.39 ± 0.52	< 0.001
TWL%	-	10.22 ± 0.27	19.15 ± 0.39	27.49 ± 0.74	< 0.001
EWL%	-	23.75 ± 0.78	44.44 ± 1.23	63.7 ± 2.06	< 0.001
EBMI%	-	23.75 ± 0.79	44.44 ± 1.23	63.7 ± 2.06	< 0.001
ΔBMI	-	4.55 ± 0.13	8.54 ± 0.19	12.27 ± 0.35	< 0.001

*BMI* Body Mass Index, *TWL%* Total weight loss Percentage, *EWL%* Excess weight loss Percentage, *EBMI%* Excess BMI loss percentage

^a^Mean ± SE

### Body composition analysis components at three time points before and after the surgery

**Fig. 1:**
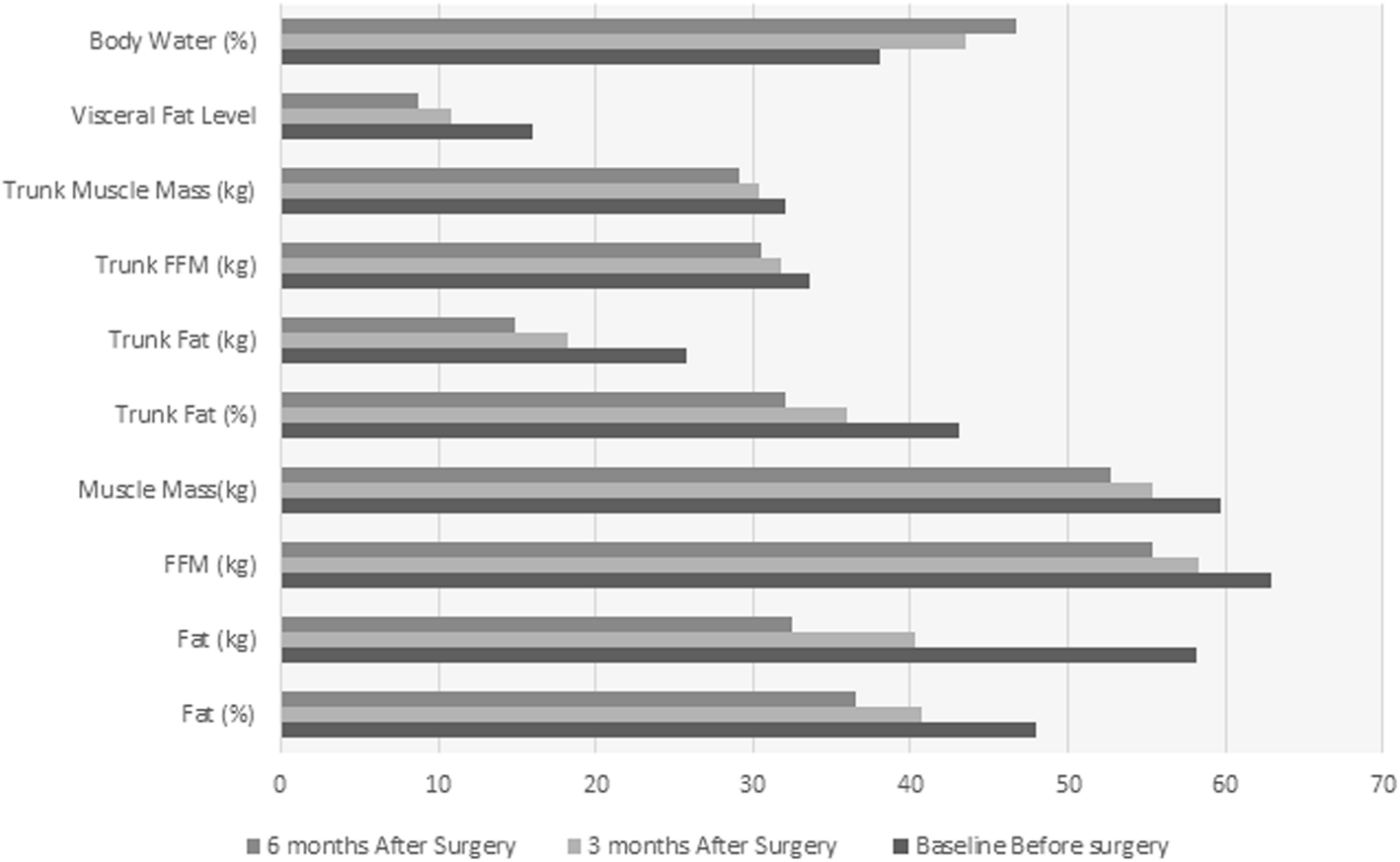
The trends of change in body composition components at three-time points before and after surgery

At three time points before and after the surgery, components of body composition analysis were evaluated, which are presented in Table 3. The patient's body composition was found to have a fat (%) of 47.96 ± 3.65% preoperatively, decreasing to 40.66 ± 0.80% and 36.46 ± 0.94% at 3 months and 6 months post-surgery, respectively. A significant drop was also noted when comparing pre-Muscle Mass (kg) 59.73 ± 13.71 kg to 3rd month Muscle Mass (kg) 55.41 ± 1.76 kg and 6 months Muscle Mass (kg) 52.66 ± 1.72 kg post-surgery, respectively (*P* < 0.001). There was a significant difference (*P* ≤ 0.001) in the variables Fat (%), Fat (kg), FFM (kg), muscle mass(kg), visceral fat level, trunk fat (%), trunk fat (kg), trunk FFM (kg), trunk muscle mass (kg) and body water over the three periods when data was recorded (Fig. 1).

**Table 3:** Body composition analysis components at three time points before and after surgery

Variables	**Before surgery**	**After Surgery**		***P*-Value**
	**Baseline**	**3 months**	**6 months**	
Fat (%)	47.96 ± 3.65	40.66 ± 0.80	36.46 ± 0.94	< 0.001
Fat (kg)	58.15 ± 13.9	40.27 ± 1.60	32.41 ± 1.40	< 0.001
FFM (kg)	62.96 ± 14.36	58.33 ± 1.84	55.36 ± 1.81	< 0.001
Muscle Mass(kg)	59.73 ± 13.71	55.41 ± 1.76	52.66 ± 1.72	< 0.001
Visceral FAT Level	15.93 ± 4.97	10.87 ± 0.50	8.67 ± 0.43	< 0.001
Trunk Fat (%)	43.05 ± 4.75	35.95 ± 0.99	32.02 ± 1.06	< 0.001
Trunk Fat (kg)	25.70 ± 6.72	18.17 ± 0.84	14.90 ± 0.78	< 0.001
Trunk FFM (kg)	33.53 ± 6.22	31.78 ± 5.98	30.45 ± 6.02	< 0.001
Trunk Muscle Mass (kg)	32.05 ± 6.00	30.39 ± 5.77	29.17 ± 5.76	< 0.001
Body Water (%)	38.07 ± 2.67	43.44 ± 0.58	46.69 ± 0.68	< 0.001

*Fat%* Fat Percentage, *FFM* Fat-free Mass

### Biochemical glycolipid profile at three time points before and after the surgery

Analysis using repeated measure ANOVA revealed that the mean serum level of PGC-1α and UCP-2 in comparison to the baseline (2.55 ± 0.04 and 5.13 ± 0.09, respectively) significantly increased at 3 (2.78 ± 0.06 and 6.11 ± 0.16, respectively) and 6 months (2.86 ± 0.23 and 8.11 ± 0.09) post-operatively (*P* = 0.01 and *P* = 0.007, respectively). The analysis of PGC-1α and UCP-2 was adjusted for the level of physical activity. (The data regarding physical activity were not included in the study but can be provided upon request.) Moreover, it showed a significant time trend decrease for HOMA-IR (*P* = 0.001), serum insulin (*P* = 0.001), FBS (*P* = 0.02), and percentage of HbA1c (*P* = 0.03) at 3- and 6-months post-surgery. Furthermore, lipid profiles as well as the liver function tests were improved at 3 and 6 months after the surgery (*P* < 0.001), except for HDL-c levels that did not change significantly post-operatively (*P* = 0.84) (Table 4).

**Table 4:** Biochemical glycolipid profile at three time points before and after surgery

Variables^a^	**Before surgery**	**After Surgery**		***P*-Value**
	**Baseline**	**3 months**	**6 months**	
PGC-1α, ng/ml	2.55 ± 0.04	2.78 ± 0.06	2.86 ± 0.23	0.01 ^b^
UCP-2, ng/ml	5.13 ± 0.09	6.11 ± 0.16	8.11 ± 0.09	0.007 ^b^
HOMA-IR	5.12 ± 0.44	2.84 ± 0.21	2.11 ± 0.23	0.001
FBS, mg/dL	109.64 ± 28.08	91.13 ± 13.87	89.53 ± 8.90	0.02
HbA1c, %	6.79 ± 0.72	5.80 ± 0.45	5.21 ± 0.39	0.03
Insulin, µIU/mL	18.07 ± 6.49	12.18 ± 4.15	9.85 ± 8.33	0.001
TG, mg/dL	163.36 ± 53.38	138.97 ± 41.46	129.81 ± 35.74	< 0.001
Chol, mg/dL	182.42 ± 40.35	168.06 ± 40.72	153.44 ± 34.98	< 0.001
LDL, mg/dL	108.61 ± 32.59	95.24 ± 28.17	86.58 ± 20.54	< 0.001
HDL, mg/dL	45.72 ± 11.86	45.00 ± 8.35	44.90 ± 2.81	0.84
ALT, IU/L	29.51 ± 12.80	23.19 ± 7.39	18.36 ± 3.97	< 0.001
AST, IU/L	31.03 ± 16.71	22.08 ± 9.21	16.08 ± 4.67	< 0.001

*PGC-1α* Peroxisome Proliferator Activated Receptor—Co-Activator-1a, *UCP-2* Uncoupling protein 2, *HOMA-IR* Homeostatic model assessment-insulin resistance, *FBS* Fasting Blood Sugar, *HbA1c* Hemoglobin A1c, *TG* triglyceride, *HDL* high-density lipoprotein, *LDL* Low-density lipoprotein, *AST* Aspartate Transaminase, *ALT* alanine aminotransferase

^a^Mean ± SE

^b^Adjusted for physical activity

### Predictive validity of the variables on pgc-1α values 6 months after the surgery

According to Table 5, the results of the multivariate linear regression analysis indicate that certain factors independently influence changes in PGC-1α levels. Specifically, FBS (B = 0.013, *P* = 0.06), insulin (B = -0.061, *P* = 0.003), uric acid (B = -0.200, *P* = 0.011), HOMA-IR (B = -0.104, *P* = 0.028), fat (kg) (B = -0.053, *P* < 0.001), and trunk fat (kg) (B = -0.083, *P* < 0.001) all contribute to the observed changes in PGC-1α. These findings suggest that an increase in insulin, HOMA-IR, uric acid, and trunk fat is associated with a less significant increase in PGC-1α.

**Table 5:** Predictive validity of the variables on PGC-1α values 6 months after surgery

Independent Variable	B (Se)^a^	β^b^	*P*-Value ^‡^
FBS, mg/dL	-0.013 (0.007)	-0.382	0.06
Insulin, µIU/mL	-0.061 (0.018)	-0.641	**0.003**
HbA1c, %	-0.01 (0.007)	-0.212	0.127
HOMA-IR	-0.104 (0.045)	-0.320	**0.028**
TG, mg/dL	-0.001 (0.002)	-0.108	0.420
Chol, mg/dL	-0.007 (0.004)	-0.617	0.115
ALT, IU/L	-0.014 (0.023)	-0.124	**0.53**
AST, IU/L	-0.013 (0.016)	-0.138	0.44
Uric Acid, g/dl	-0.200 (0.075)	-0.375	**0.011**
Fat, kg	-0.053 (0.012)	-1.109	**0.000**
Trunk Fat, kg	-0.083 (0.021)	-0.964	**0.000**

*PGC-1α* Peroxisome Proliferator- Activated Receptor—Co-Activator-1a, *HOMA-IR* homeostatic model assessment-insulin resistance, *FBS* Fasting Blood Sugar, *HbA1c* Hemoglobin A1c, *TG* triglyceride, *HDL* high-density lipoprotein, *LDL* Low-density lipoprotein, *AST* Aspartate Transaminase, *ALT* alanine aminotransferase

Model *P*-value: 0.001, R: 0.76, R-square: 0.58, Adjusted R Square: 0.45

^a^Unstandardized coefficient

^b^Standardized estimated coefficient

^‡^Multiple hierarchical linear regression is used

### Predictive validity of the variables on UCP-2 values 6 months after the surgery

According to Table 6, the results of the multivariate linear regression analysis showed that TSH (B = -0.312, *P* = 0.026), AST (B = -0.087, *P* = 0.009), Fat (%) (B = -0.410 (0.116), *P* = 0.001), Fat (kg) (B = -0.295, *P* = 0.001), and FFM (B = 0.131, *P* = 0.004) were identified as significant predictive variables in the changing trend of UCP-2 level.

**Table 6:** Predictive validity of the variables on UCP-2 values 6 months after surgery

Independent Variable	**B (Se)**^**a**^	**β**^**b**^	*P*-Value^**‡**^
FBS, mg/dL	-0.021 (0.023)	-0.021	0.388
Insulin, µIU/mL	-0.039 (0.036)	-0.167	0.293
HbA1c, %	-0.420 (0.476)	-0.420	0.386
HOMA-IR	-0.082 (0.10)	-0.117	0.049
TG, mg/dL	-0.001 (0.005)	-0.031	0.865
LDL, mg/dL	-0.009 (0.006)	-0.244	0.106
HDL, mg/dL	0.028 (0.060)	0.085	0.637
ALT, IU/L	-0.026 (0.051)	-0.091	0.019
AST, IU/L	-0.087 (0.031)	-0-.395	**0.009**
Uric Acid, gr/dl	-0.093 (0.231)	-0.072	0.691
TSH, mIU/L	-0.312 (0.133)	-0.333	**0.026**
Fat, %	-0.410 (0.116)	-2.363	**0.001**
Fat, kg	-0.295 (0.083)	-2.537	**0.001**
FFM, kg	0.131 (0.042)	1.453	**0.004**

*UCP-2* Uncoupling protein -2, *HOMA-IR* homeostatic model assessment-insulin resistance, *FBS* Fasting Blood Sugar, *HbA1c* Hemoglobin A1c, *TG* triglyceride, *HDL* high-density lipoprotein, *LDL* Low-density lipoprotein, *AST* Aspartate Transaminase, *ALT* alanine aminotransferase

Model *P*-value: 0.006, R: 0.69, R-square: 0.48, Adjusted R Square: 0.34

^a^Unstandardized coefficient

^b^Standardized estimated coefficient

^‡^Multiple hierarchical linear regression is used

## Discussion

Bariatric surgery is considered an effective way for accomplishing appropriate weight loss, both in the short-term and long-term in those with severe obesity. Further, it can facilitate the control and prevention of obesity-related co-morbidities [1, 2]. One of the main mechanisms concerning obesity or lack of success in weight reduction in patients with morbid obesity is mitochondrial dysfunction [6].

In the present study, significant decreases were found regarding weight, BMI, body fat, muscle mass, trunk muscle mass, visceral fat, and trunk fat, and an increase was seen in body water in the patients undergoing bariatric surgery overtime after 3, and 6 months post-operatively. These findings confirm the results of a previous study done by Maïmoun et al. [14], that assessed the changes in body weight and composition after bariatric surgery in 1 and 12 months post-surgery. They also reported significant decreases in body weight, fat, lean body mass, and visceral fat, especially in the long term after the surgery [14]. This was also in line with the results of the study by Sivakumar and colleagues that showed a decreasing trend in body weight, BMI, body fat, and muscle mass 12 months after the surgery [15]**.**

As another finding of the present study, assessment of biochemical glycolipid profile at three time points before and after the surgery, showed significant increasing trends in the serum levels of PGC-1α and UCP-2 over the time, post-operatively (3 and 6 months) in comparison to the baseline. No study assessed the changes in PGC-1α or UCP-2 post-operatively and only one animal study emphasized the increase in the levels of AMPK [16] as an important inducer of PGC-1α [9] which could indirectly confirm the increasing trend in the PGC-1α after the surgery.

Moreover, significant time trend decreases for HOMA-IR, serum Insulin, FBS, and percentage of HbA1c were observed at 3 and 6 months post-operatively. In addition, improvements in lipid profiles and liver function tests were seen at 3 and 6 months after the surgery. This finding was also observed in a previous study by Liu and colleagues who observed reduced levels of insulin, HbA1c, and blood glucose post-operatively in those undergoing bariatric surgery [17]. Moreover, the decreases in the liver enzymes were in accordance with a previous study showing post-operative reduction of AST and ALT, 2 years after the surgery. It was asserted that the increased level of ALT is related to high serum glucose, low insulin sensitivity, and diabetes risk [18]. Hence, the decrease in ALT and AST levels post-operatively can be a possible mechanism describing the simultaneous decrease of liver enzymes, FBS, and HOMA-IR.

Several mechanisms are responsible for the decrease in biochemical parameters after bariatric surgery. One of the main mechanisms, independent of weight loss effects, is pertinent to the changes in the gut microbiome. The level of circulating bile acids is increased after the bariatric surgery and this can change the gut microbiome and this can affect the decrease in the liver enzymes independent of the weight-loss effects of the bariatric surgery [19–21]. Moreover, any increase in the activity of the PPAR pathway could definitely decrease the level of liver enzymes [22] and this was seen in the present study regarding the increase in PGC-1α which probably affected the levels of liver enzymes post-operatively.

According to further analysis, it was observed that FBS, insulin, uric acid, HOMA-IR, fat (kg), and trunk fat (kg) have independent prognostic values for PGC-1α level which means that their decrease was accompanied by and related to the increase in the PGC-1α level post-operatively.

As another finding, TSH, AST, fat, and FFM had independent predictive effects on the level of UCP-2. This means that higher levels of UCP-2 were accompanied by increased FFM and decreased TSH and body fat. Furthermore, these factors could predict the changes in UCP-2 after bariatric surgery. This finding confirms a previous report in a systematic review that mentioned how changes in the expression of UCP-2 and UCP-3 could affect weight loss after surgery [23]. Therefore, it emphasizes the relationship between increased levels of UCP-2 and a decrease in fat mass and overall weight.

Regulatory mechanisms of mitochondria are involved in obesity and its pathogenesis and the effects of calorie restriction. In this regard, PGC-1α is definitely related to the metabolic status in the white adipose tissue [23] and any increase in the PGC-1α and subsequently UCP-2 in the white adipose tissue shows the feature of the brown adipose tissue as they can induce fat oxidation and thermogenesis that all help weight loss and increase insulin sensitivity [11]. As a fact, PGC-1α can affect the neuro-metabolism, especially the orexinergic system [24]. As a protein interacting with PPAR-γ, PGC-1α [25] could bind to some transcription factors such as estrogen-related receptor α (ERRα), nuclear respiratory factor (NRF)-1, and NRF-2, and this way it can activate some genes [26–29]. NRF-1 and NRF-2 could affect some mitochondrial genes, especially those that are active in the respiratory chain [28]. ERRα can regulate the metabolic pathways related to the tricarboxylic acid cycle and β-oxidation and mitochondrial metabolism [29–31], showing the effects of PGC-1α in the fat metabolism which is necessary in weight management. In addition, UCP-2 is a key regulator of energy expenditure in the mitochondria and they have a pivotal role in the management of obesity, hyperinsulinemia, and metabolic syndrome [32]. UCP-2, as a protein expressed in the adipose tissue and pancreatic endocrine system [33], could modulate insulin and glucose metabolism and regulate insulin secretion which could relate it to obesity and diabetes [34, 35]. Moreover, the effect of PGC-1α on the modulation of the insulin pathway was previously observed [36]. These effects could possibly justify the simultaneous decrease in body weight and body composition, HOMA-IR, serum insulin, FBS, and percentage of HbA1c with the increase in UCP-2 and PGC-1α in the present study. This increase could show the protective effects of UCP-2 and PGC-1α on energy, fat, and glucose metabolism. However, another hypothesis can also be considered that the increase in energy expenditure serum markers may result from the reversal of comorbidities, thus providing an additional explanation for the results.

On the other hand, it is mentioned previously that the decrease in the adipose tissue following the bariatric surgery could decrease the TSH level subsequently due to the decreased expression of TSH genes or TSH receptor genes [37]. Hence, it can be hypothesized that the increase in the UCP-2 after the surgery and its effects on decreasing body fat and weight, could indirectly modulate thyroid hormones and decrease TSH and this can in part explain the prognostic significance of TSH for UCP-2 observed in the current study.

Previous studies illustrated that PGC-1α can affect various metabolic pathways or medical conditions including diabetes, neurodegenerative diseases, obesity, and the like [24]. Hence, the increase shown in the levels of PGC-1α and UCP-2 could possibly correlate to the changes in weight and appetite due to the metabolic effects of PGC-1α and UCP-2, especially in the regulation of fat metabolism and energy expenditure in the mitochondria. This increase could be a possible underling mechanism show the beneficial relationship between weight loss and metabolism through changes in PGC-1α and UCP-2.

This study had some strengths from which the most important one is related to the complete assessment of the patients pre- and post-operatively over various times in six-month periods after surgery, which the most changes in weight, body composition, and metabolic parameters occurred in the first six months postoperatively. Furthermore, the present study evaluates the most important cellular regulator of mitochondrial energy homeostasis, and energy expenditure indicators and their relationship with body composition at three time points pre- and post- bariatric surgery. However, the present study had some limitations as well. Due to the difficulties in following the patients in the long term, only six months of follow-up was possible for the present study compared to other studies with longer follow-ups. Surely, it would be better to follow the patients for longer durations to assess any chance of recurrence of any abnormality. Additionally, due to the design of the study, it was not possible to establish causal relationships. Moreover, due to financial constraints and a limited research budget, we were only able to measure the body composition and blood biochemical parameters at three stages and in larger sample size.

## Conclusion

To sum up, patients who undergo bariatric surgeries may experience significant reductions in weight, BMI, body fat, FFM, insulin, FBS, HbA1C, HOMA-IR, blood lipids, and liver enzymes after time, post-operatively. Moreover, increase in UCP-2 and PGC-1α observed post-surgery could be connected to these changes. The effects of UCP-2 on insulin/glucose regulation, fat metabolism, energy expenditure, liver enzyme modulation, and its indirect impact on TSH gene expression (due to fat loss) may explain some of the aforementioned outcomes. Moreover, the effects of PGC-1α on glucose metabolism, fat oxidation, and weight management through the regulation of mitochondrial metabolism may also contribute to these changes. However, additional research is needed to fully comprehend the exact mechanisms and potential cause-and-effect relationship between changes in energy expenditure, factors influencing thermogenesis, and post-surgical metabolic outcomes. It is important for these studies to be conducted over a prolonged period to ensure a thorough understanding.

## Data Availability

All data generated or analyzed during this study are included in this article. Further inquiries can be directed to the corresponding author.
